# Hemi(4,4′-bipyridinium) hexa­fluorido­phosphate bis­(4-amino­benzoic acid) 4,4′-bipyridine monohydrate

**DOI:** 10.1107/S1600536808043900

**Published:** 2009-01-10

**Authors:** Yi-Yi Wu, Chun-De Huang, Jie-Xuan Huang, Rong-Hua Zeng, Yi-Fan Luo

**Affiliations:** a School of Chemistry and Environment, South China Normal University, Guangzhou 510006, People’s Republic of China; b Key Laboratory of Electrochemical Technology on Energy Storage and Power Generation in Guangdong Universities, Guangzhou 510631, People’s Republic of China

## Abstract

In the title compound, 0.5C_10_H_10_N_2_^2+^·PF_6_^−^·C_10_H_8_N_2_·2C_7_H_7_NO_2_·H_2_O, the cation is located on a center of symmetry. The crystal structure is determined by a complex three-dimensional network of inter­molecular O—H⋯O, O—H⋯N, N—H⋯N and N—H⋯F hydrogen bonds. π–π stacking inter­actions between neighboring pyridyl rings are also present; the centroid–centroid distance is 3.643 (5) Å. The hexa­fluoridophosphate anion is disordered over two positions with site-occupancy factors of *ca* 0.6 and 0.4.

## Related literature

For the use of 4-amino­benzoic acid and 4,4′-bipyridine for the construction of three-dimensional network motifs, see: Hu *et al.* (2003 ▶); Yang *et al.* (2004 ▶).
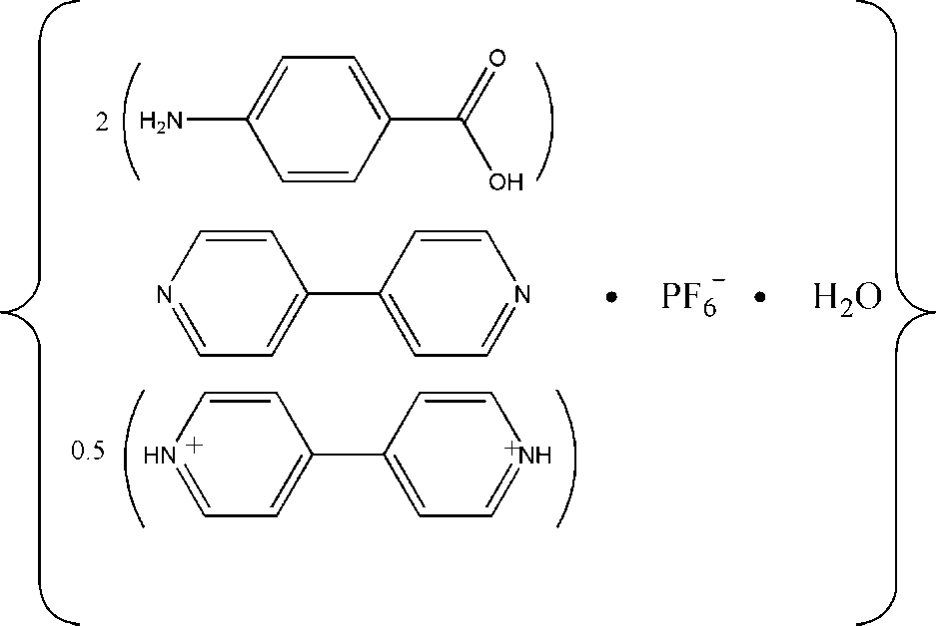


## Experimental

### 

#### Crystal data

0.5C_10_H_10_N_2_^2+^·PF_6_^−^·C_10_H_8_N_2_·2C_7_H_7_NO_2_·H_2_O
*M* *_r_* = 672.54
Triclinic, 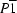

*a* = 10.1032 (2) Å
*b* = 10.1142 (2) Å
*c* = 16.8906 (3) Å
α = 92.557 (1)°
β = 98.063 (1)°
γ = 117.346 (1)°
*V* = 1506.23 (5) Å^3^
*Z* = 2
Mo *K*α radiation
μ = 0.18 mm^−1^
*T* = 296 (2) K
0.18 × 0.15 × 0.14 mm

#### Data collection

Bruker APEXII area-detector diffractometer
Absorption correction: none
22618 measured reflections
7121 independent reflections
3129 reflections with *I* > 2σ(*I*)
*R*_int_ = 0.057

#### Refinement

*R*[*F* ^2^ > 2σ(*F* ^2^)] = 0.066
*wR*(*F* ^2^) = 0.205
*S* = 1.02
7121 reflections
454 parameters
43 restraints
H-atom parameters constrained
Δρ_max_ = 0.29 e Å^−3^
Δρ_min_ = −0.34 e Å^−3^

### 

Data collection: *APEX2* (Bruker, 2004 ▶); cell refinement: *APEX2* and *SAINT* (Bruker, 2004 ▶); data reduction: *SAINT*; program(s) used to solve structure: *SHELXS97* (Sheldrick, 2008 ▶); program(s) used to refine structure: *SHELXL97* (Sheldrick, 2008 ▶); molecular graphics: *SHELXTL* (Sheldrick, 2008 ▶); software used to prepare material for publication: *SHELXL97*.

## Supplementary Material

Crystal structure: contains datablocks I, global. DOI: 10.1107/S1600536808043900/wn2299sup1.cif

Structure factors: contains datablocks I. DOI: 10.1107/S1600536808043900/wn2299Isup2.hkl

Additional supplementary materials:  crystallographic information; 3D view; checkCIF report

## Figures and Tables

**Table 1:** Hydrogen-bond geometry (Å, °)

*D*—H⋯*A*	*D*—H	H⋯*A*	*D*⋯*A*	*D*—H⋯*A*
O1*W*—H2*W*⋯O4^i^	0.89	1.87	2.751 (3)	169
O1*W*—H1*W*⋯O2^ii^	0.79	2.01	2.799 (3)	174
O3—H3*A*⋯N3^iii^	0.82	1.87	2.686 (3)	174
O1—H1⋯O1*W*^iv^	0.82	1.80	2.617 (3)	173
N1—H1*B*⋯F3	0.86	2.57	3.324 (4)	147
N5—H27⋯N4	0.86	1.84	2.700 (4)	176

Symmetry codes: (i) 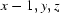; (ii) 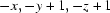; (iii) 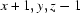; (iv) 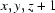.

**Fig. 1.**
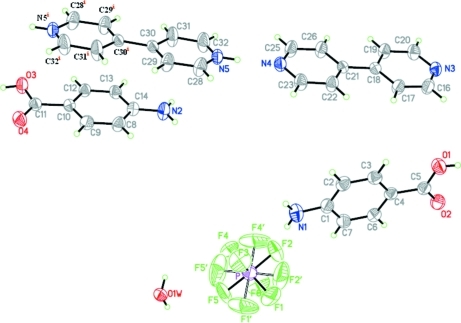
The molecular structure showing the atomic-numbering scheme. Displacement ellipsoids are drawn at the 30% probability level. Hydrogen atoms are shown as spheres of arbitrary radius. Symmetry code: (i) 2 - x, -y, 1 - z.

**Fig. 2.**
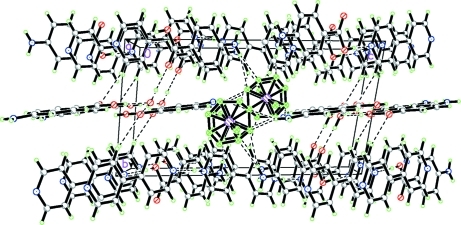
A view of the three-dimensional supramolecular network. Hydrogen bonds are shown as dashed lines.

## supplementary crystallographic information

### Comment

Hydrogen-bonding interactions between ligands are specific and directional. In this context, 4-aminobenzoic acid and 4,4'-bipyridine are excellent candidates for the construction of three-dimensional network motifs, which form regular hydrogen bonds, functioning as both hydrogen-bond donor and acceptor (Hu *et al.*, 2003; Yang *et al.*, 2004). Recently, we obtained the title compound under hydrothermal conditions and report its crystal structure here.

In the title compound (Fig. 1), all bond lengths and angles are unexceptional. The 4,4'-bipyridinium cation, which is located on a center of symmetry, is, on both sides, a hydrogen bond donor to unprotonated molecules of 4,4'-bipyridine. These are, in turn, hydrogen bond acceptors for one of the two independent molecules of 4-aminobenzoic acid. Thus the two 4-aminobenzoic acid molecules, the 4,4'-bipyridine and 4,4'-bipyridinium cation are connected by hydrogen bonding interactions to form a linear centrosymmetric chain. Further each of these chains is connected with each of two other chains by O—H···O hydrogen bonds (Table 1), *via* the interstitial solvent water molecules and the other independent 4-aminobenzoic acid molecules, forming, in effect, infinite chains with each repeating unit of five molecules offset against the next unit by the width of the connecting (C_7_NH_7_O_2_)_2_.(H_2_O)_2_ unit. π-π stacking interactions (the centroid-centroid distance between neighboring pyridyl rings is 3.643 Å) connect these chains to produce a three-dimensional network motif (Fig. 2).

### Experimental

4-Aminobenzoic acid (1 mmol, 0.137 g), 4,4'-bipyridine (1 mmol, 0.156 g) and sodium hexafluoridophosphate (1 mmol, 0.162 g) were dissolved in hot water with stirring. Colorless single crystals were obtained at room temperature by slow evaporation of the solvent over a period of several days.

### Refinement

The disordered perchlorate ion was refined over two sites, with refined occupancies of 0.625 (8) and 0.375 (9). The P···F and F···F distances were restrained to be 1.48 (1) and 2.10 (3) Å, respectively. Water H atoms were located in a difference Fourier map and refined with distance restraints of O—H = 0.86 Å and H···H = 1.39 Å. The H atom bound to the N5 nitrogen atom in the cation and the carboxylic H atoms were refined with distance restraints of N—H = 0.90 Å and O—H = 0.90 Å, respectively. All other H atoms were placed at calculated positions and treated as riding on the parent atoms, with C—H = 0.93 Å, N—H = 0.86 Å, and with *U*_iso_(H) = 1.2*U*_eq_(C, N).

### Crystal data

**Table:**

0.5C_10_H_10_N_2_^2+^·PF_6_^−^·C_10_H_8_N_2_·2C_7_H_7_NO_2_·H_2_O	*Z* = 2
*M**_r_* = 672.54	*F*(000) = 694
Triclinic, *P*1	*D*_x_ = 1.483 Mg m^−^^3^
Hall symbol: -P 1	Mo *K*α radiation, λ = 0.71073 Å
*a* = 10.1032 (2) Å	Cell parameters from 2023 reflections
*b* = 10.1142 (2) Å	θ = 2.4–19.5°
*c* = 16.8906 (3) Å	µ = 0.18 mm^−^^1^
α = 92.557 (1)°	*T* = 296 K
β = 98.063 (1)°	Block, colourless
γ = 117.346 (1)°	0.18 × 0.15 × 0.14 mm
*V* = 1506.23 (5) Å^3^	

### Data collection

**Table:**

Bruker APEXII area-detector diffractometer	3129 reflections with *I* > 2σ(*I*)
Radiation source: fine-focus sealed tube	*R*_int_ = 0.057
graphite	θ_max_ = 27.9°, θ_min_ = 2.3°
φ and ω scans	*h* = −13→13
22618 measured reflections	*k* = −13→13
7121 independent reflections	*l* = −20→22

### Refinement

**Table:**

Refinement on *F*^2^	Secondary atom site location: difference Fourier map
Least-squares matrix: full	Hydrogen site location: inferred from neighbouring sites
*R*[*F*^2^ > 2σ(*F*^2^)] = 0.066	H-atom parameters constrained
*wR*(*F*^2^) = 0.205	*w* = 1/[σ^2^(*F*_o_^2^) + (0.0875*P*)^2^ + 0.1722*P*] where *P* = (*F*_o_^2^ + 2*F*_c_^2^)/3
*S* = 1.02	(Δ/σ)_max_ = 0.006
7121 reflections	Δρ_max_ = 0.29 e Å^−^^3^
454 parameters	Δρ_min_ = −0.33 e Å^−^^3^
43 restraints	Extinction correction: *SHELXL97* (Sheldrick, 2008)
Primary atom site location: structure-invariant direct methods	Extinction coefficient: 0.009 (2)

### Special details

**Table:**

Geometry. All e.s.d.'s (except the e.s.d. in the dihedral angle between two l.s. planes) are estimated using the full covariance matrix. The cell e.s.d.'s are taken into account individually in the estimation of e.s.d.'s in distances, angles and torsion angles; correlations between e.s.d.'s in cell parameters are only used when they are defined by crystal symmetry. An approximate (isotropic) treatment of cell e.s.d.'s is used for estimating e.s.d.'s involving l.s. planes.
Refinement. Refinement of *F*^2^ against ALL reflections. The weighted *R*-factor *wR* and goodness of fit *S* are based on *F*^2^, conventional *R*-factors *R* are based on *F*, with *F* set to zero for negative *F*^2^. The threshold expression of *F*^2^ > σ(*F*^2^) is used only for calculating *R*-factors(gt) *etc*. and is not relevant to the choice of reflections for refinement. *R*-factors based on *F*^2^ are statistically about twice as large as those based on *F*, and *R*- factors based on ALL data will be even larger.

### Fractional atomic coordinates and isotropic or equivalent isotropic displacement parameters (Å^2^)

**Table:**

	*x*	*y*	*z*	*U*_iso_*/*U*_eq_	Occ. (<1)
C1	0.4034 (4)	0.5337 (4)	0.7188 (2)	0.0650 (9)	
C2	0.4778 (4)	0.5250 (4)	0.7922 (2)	0.0656 (9)	
H2	0.5740	0.5331	0.7960	0.079*	
C3	0.4122 (4)	0.5047 (4)	0.8589 (2)	0.0602 (9)	
H3	0.4645	0.4991	0.9075	0.072*	
C4	0.2682 (3)	0.4922 (3)	0.8557 (2)	0.0523 (8)	
C5	0.1957 (4)	0.4726 (3)	0.9266 (2)	0.0576 (8)	
C6	0.1930 (4)	0.4994 (3)	0.7822 (2)	0.0590 (8)	
H6	0.0961	0.4896	0.7783	0.071*	
C7	0.2589 (4)	0.5208 (4)	0.7152 (2)	0.0646 (9)	
H7	0.2068	0.5267	0.6666	0.077*	
C8	0.8336 (4)	0.1574 (4)	0.3481 (2)	0.0662 (9)	
H8	0.8482	0.2431	0.3790	0.079*	
C9	0.9268 (4)	0.1700 (4)	0.2940 (2)	0.0627 (9)	
H9	1.0027	0.2642	0.2880	0.075*	
C10	0.9095 (3)	0.0437 (3)	0.24786 (18)	0.0503 (7)	
C11	1.0119 (4)	0.0620 (4)	0.19048 (19)	0.0555 (8)	
C12	0.7954 (3)	−0.0956 (4)	0.25815 (19)	0.0545 (8)	
H12	0.7825	−0.1815	0.2283	0.065*	
C13	0.7011 (4)	−0.1079 (4)	0.31200 (19)	0.0579 (8)	
H13	0.6254	−0.2022	0.3180	0.070*	
C14	0.7174 (4)	0.0183 (4)	0.35751 (19)	0.0578 (8)	
N3	0.1693 (3)	−0.0235 (3)	1.04268 (16)	0.0592 (7)	
C16	0.2657 (5)	0.1119 (4)	1.0271 (2)	0.0754 (11)	
H16	0.2693	0.1953	1.0549	0.090*	
C17	0.3601 (4)	0.1349 (4)	0.9723 (2)	0.0682 (10)	
H17	0.4246	0.2321	0.9634	0.082*	
C18	0.3597 (3)	0.0145 (3)	0.93018 (17)	0.0464 (7)	
C19	0.2589 (3)	−0.1253 (4)	0.9462 (2)	0.0606 (9)	
H19	0.2522	−0.2109	0.9192	0.073*	
C20	0.1679 (4)	−0.1388 (4)	1.0023 (2)	0.0650 (9)	
H20	0.1018	−0.2346	1.0122	0.078*	
C21	0.4624 (3)	0.0328 (3)	0.87181 (17)	0.0452 (7)	
C22	0.5692 (4)	0.1715 (4)	0.8566 (2)	0.0641 (9)	
H22	0.5793	0.2585	0.8839	0.077*	
C23	0.6600 (4)	0.1817 (4)	0.8017 (2)	0.0662 (9)	
H23	0.7302	0.2765	0.7925	0.079*	
N4	0.6522 (3)	0.0623 (3)	0.76098 (15)	0.0576 (7)	
C25	0.5513 (5)	−0.0691 (4)	0.7747 (2)	0.0791 (11)	
H25	0.5432	−0.1540	0.7460	0.095*	
C26	0.4564 (4)	−0.0888 (4)	0.8291 (2)	0.0734 (11)	
H26	0.3877	−0.1852	0.8369	0.088*	
N5	0.8058 (3)	0.0432 (3)	0.64624 (16)	0.0600 (7)	
H27	0.7588	0.0538	0.6827	0.072*	
C28	0.9248 (4)	0.1591 (4)	0.6303 (2)	0.0761 (11)	
H28	0.9572	0.2528	0.6586	0.091*	
C29	1.0029 (4)	0.1461 (4)	0.5730 (2)	0.0719 (10)	
H29	1.0865	0.2306	0.5628	0.086*	
C30	0.9587 (3)	0.0097 (3)	0.53068 (17)	0.0481 (7)	
C31	0.8329 (5)	−0.1090 (4)	0.5490 (3)	0.0920 (14)	
H31	0.7977	−0.2044	0.5220	0.110*	
C32	0.7592 (5)	−0.0883 (5)	0.6064 (3)	0.0932 (13)	
H32	0.6736	−0.1700	0.6172	0.112*	
F3	0.4396 (3)	0.6418 (3)	0.46460 (18)	0.1343 (10)	
F6	0.0964 (3)	0.5330 (3)	0.3961 (2)	0.1465 (12)	
N1	0.4710 (4)	0.5607 (4)	0.6526 (2)	0.0960 (11)	
H1A	0.5617	0.5727	0.6560	0.115*	
H1B	0.4230	0.5658	0.6076	0.115*	
N2	0.6215 (3)	0.0068 (4)	0.41031 (18)	0.0812 (9)	
H2A	0.5499	−0.0797	0.4156	0.097*	
H2B	0.6331	0.0860	0.4380	0.097*	
O1	0.2766 (3)	0.4659 (3)	0.99380 (15)	0.0756 (7)	
H1	0.2309	0.4589	1.0312	0.113*	
O2	0.0709 (3)	0.4645 (3)	0.92571 (15)	0.0767 (7)	
O3	0.9955 (3)	−0.0651 (3)	0.15538 (15)	0.0728 (7)	
H3A	1.0465	−0.0476	0.1197	0.109*	
O4	1.1065 (3)	0.1827 (3)	0.17604 (15)	0.0772 (7)	
P	0.26740 (12)	0.58756 (12)	0.43141 (7)	0.0774 (4)	
O1W	0.1528 (2)	0.4502 (3)	0.12153 (13)	0.0703 (7)	
H1W	0.0862	0.4708	0.1110	0.105*	
H2W	0.1255	0.3604	0.1373	0.105*	
F1	0.2698 (15)	0.7215 (12)	0.4737 (9)	0.129 (5)	0.375 (11)
F2	0.2105 (13)	0.4994 (17)	0.5008 (6)	0.125 (5)	0.375 (11)
F4	0.2560 (8)	0.4439 (8)	0.3880 (8)	0.090 (4)	0.375 (11)
F5	0.3176 (16)	0.6648 (13)	0.3621 (5)	0.139 (5)	0.375 (11)
F1'	0.3113 (7)	0.7452 (10)	0.4047 (10)	0.192 (6)	0.625 (11)
F4'	0.2424 (12)	0.4441 (8)	0.4616 (10)	0.222 (6)	0.625 (11)
F5'	0.2826 (14)	0.534 (2)	0.3497 (4)	0.239 (7)	0.625 (11)
F2'	0.2667 (13)	0.6511 (18)	0.5141 (5)	0.217 (5)	0.625 (11)

### Atomic displacement parameters (Å^2^)

**Table:**

	*U*^11^	*U*^22^	*U*^33^	*U*^12^	*U*^13^	*U*^23^
C1	0.070 (2)	0.051 (2)	0.073 (2)	0.0229 (18)	0.030 (2)	0.0092 (17)
C2	0.056 (2)	0.062 (2)	0.084 (3)	0.0283 (18)	0.0231 (19)	0.0113 (19)
C3	0.0564 (19)	0.055 (2)	0.070 (2)	0.0254 (16)	0.0149 (17)	0.0129 (17)
C4	0.0520 (18)	0.0403 (18)	0.064 (2)	0.0199 (15)	0.0164 (16)	0.0088 (15)
C5	0.059 (2)	0.0442 (19)	0.069 (2)	0.0215 (16)	0.0211 (18)	0.0112 (16)
C6	0.0518 (18)	0.051 (2)	0.070 (2)	0.0217 (16)	0.0105 (17)	0.0028 (17)
C7	0.068 (2)	0.057 (2)	0.067 (2)	0.0268 (18)	0.0146 (18)	0.0086 (17)
C8	0.071 (2)	0.056 (2)	0.072 (2)	0.0297 (19)	0.0200 (19)	−0.0035 (18)
C9	0.062 (2)	0.049 (2)	0.072 (2)	0.0206 (17)	0.0181 (18)	0.0064 (17)
C10	0.0495 (17)	0.051 (2)	0.0510 (18)	0.0224 (15)	0.0134 (14)	0.0095 (15)
C11	0.0519 (18)	0.061 (2)	0.055 (2)	0.0264 (18)	0.0116 (16)	0.0127 (17)
C12	0.0565 (19)	0.0490 (19)	0.057 (2)	0.0225 (16)	0.0166 (16)	0.0052 (15)
C13	0.0565 (19)	0.049 (2)	0.061 (2)	0.0174 (16)	0.0175 (16)	0.0089 (16)
C14	0.0563 (19)	0.062 (2)	0.056 (2)	0.0272 (18)	0.0155 (16)	0.0050 (17)
N3	0.0575 (16)	0.069 (2)	0.0549 (17)	0.0288 (15)	0.0229 (13)	0.0115 (15)
C16	0.099 (3)	0.066 (3)	0.080 (3)	0.045 (2)	0.045 (2)	0.014 (2)
C17	0.085 (3)	0.048 (2)	0.080 (2)	0.0299 (19)	0.045 (2)	0.0172 (18)
C18	0.0454 (16)	0.0493 (19)	0.0436 (17)	0.0213 (15)	0.0086 (13)	0.0071 (14)
C19	0.0590 (19)	0.046 (2)	0.070 (2)	0.0144 (16)	0.0276 (17)	0.0035 (16)
C20	0.056 (2)	0.061 (2)	0.068 (2)	0.0151 (17)	0.0243 (17)	0.0088 (18)
C21	0.0466 (16)	0.0461 (18)	0.0424 (17)	0.0200 (14)	0.0114 (13)	0.0086 (13)
C22	0.072 (2)	0.047 (2)	0.068 (2)	0.0196 (17)	0.0305 (18)	0.0026 (16)
C23	0.067 (2)	0.053 (2)	0.068 (2)	0.0147 (17)	0.0289 (18)	0.0063 (18)
N4	0.0616 (16)	0.0588 (18)	0.0543 (17)	0.0268 (15)	0.0213 (13)	0.0086 (14)
C25	0.105 (3)	0.052 (2)	0.089 (3)	0.034 (2)	0.053 (2)	0.008 (2)
C26	0.089 (3)	0.042 (2)	0.088 (3)	0.0195 (18)	0.054 (2)	0.0121 (18)
N5	0.0648 (18)	0.070 (2)	0.0529 (16)	0.0334 (16)	0.0259 (14)	0.0092 (15)
C28	0.069 (2)	0.063 (2)	0.087 (3)	0.021 (2)	0.030 (2)	−0.011 (2)
C29	0.060 (2)	0.056 (2)	0.085 (3)	0.0109 (17)	0.0320 (19)	−0.0064 (19)
C30	0.0546 (18)	0.0451 (18)	0.0462 (17)	0.0219 (15)	0.0172 (14)	0.0127 (15)
C31	0.122 (3)	0.048 (2)	0.104 (3)	0.023 (2)	0.078 (3)	0.008 (2)
C32	0.110 (3)	0.060 (3)	0.104 (3)	0.022 (2)	0.069 (3)	0.013 (2)
F3	0.0937 (19)	0.140 (3)	0.145 (3)	0.0434 (17)	−0.0065 (17)	−0.0012 (19)
F6	0.0795 (18)	0.118 (2)	0.226 (4)	0.0400 (16)	0.007 (2)	0.001 (2)
N1	0.104 (3)	0.109 (3)	0.090 (3)	0.052 (2)	0.051 (2)	0.029 (2)
N2	0.079 (2)	0.077 (2)	0.085 (2)	0.0292 (17)	0.0385 (18)	−0.0015 (17)
O1	0.0770 (17)	0.0873 (18)	0.0663 (16)	0.0383 (15)	0.0222 (13)	0.0198 (14)
O2	0.0705 (16)	0.0926 (19)	0.0860 (18)	0.0477 (15)	0.0338 (14)	0.0260 (14)
O3	0.0778 (17)	0.0681 (17)	0.0758 (17)	0.0300 (13)	0.0393 (13)	0.0101 (13)
O4	0.0750 (16)	0.0637 (17)	0.0926 (19)	0.0245 (14)	0.0391 (14)	0.0237 (14)
P	0.0796 (7)	0.0646 (7)	0.0843 (8)	0.0286 (6)	0.0226 (6)	0.0093 (6)
O1W	0.0699 (15)	0.0638 (15)	0.0736 (16)	0.0265 (12)	0.0180 (12)	0.0168 (12)
F1	0.171 (7)	0.073 (6)	0.130 (8)	0.054 (5)	0.012 (6)	−0.041 (5)
F2	0.122 (6)	0.140 (9)	0.098 (6)	0.035 (6)	0.057 (4)	0.047 (6)
F4	0.067 (4)	0.062 (5)	0.135 (8)	0.031 (3)	0.009 (5)	−0.014 (4)
F5	0.193 (8)	0.108 (8)	0.080 (6)	0.026 (6)	0.071 (5)	0.038 (5)
F1'	0.100 (4)	0.124 (7)	0.341 (14)	0.033 (4)	0.045 (6)	0.138 (8)
F4'	0.223 (9)	0.082 (5)	0.352 (18)	0.066 (5)	0.023 (10)	0.089 (8)
F5'	0.241 (11)	0.45 (2)	0.085 (5)	0.228 (14)	−0.006 (5)	−0.058 (9)
F2'	0.294 (11)	0.263 (14)	0.141 (7)	0.164 (11)	0.081 (7)	−0.032 (8)

### Geometric parameters (Å, °)

**Table:**

C1—N1	1.364 (4)	C19—C20	1.380 (4)
C1—C2	1.387 (5)	C19—H19	0.9300
C1—C7	1.396 (5)	C20—H20	0.9300
C2—C3	1.361 (5)	C21—C26	1.370 (4)
C2—H2	0.9300	C21—C22	1.385 (4)
C3—C4	1.394 (4)	C22—C23	1.368 (4)
C3—H3	0.9300	C22—H22	0.9300
C4—C6	1.385 (4)	C23—N4	1.327 (4)
C4—C5	1.465 (4)	C23—H23	0.9300
C5—O2	1.223 (4)	N4—C25	1.306 (4)
C5—O1	1.325 (4)	C25—C26	1.376 (5)
C6—C7	1.368 (4)	C25—H25	0.9300
C6—H6	0.9300	C26—H26	0.9300
C7—H7	0.9300	N5—C32	1.304 (4)
C8—C9	1.372 (5)	N5—C28	1.310 (4)
C8—C14	1.392 (5)	N5—H27	0.8600
C8—H8	0.9300	C28—C29	1.369 (5)
C9—C10	1.391 (4)	C28—H28	0.9300
C9—H9	0.9300	C29—C30	1.367 (4)
C10—C12	1.389 (4)	C29—H29	0.9300
C10—C11	1.472 (4)	C30—C31	1.377 (5)
C11—O4	1.218 (4)	C30—C30^i^	1.477 (6)
C11—O3	1.318 (4)	C31—C32	1.366 (5)
C12—C13	1.376 (4)	C31—H31	0.9300
C12—H12	0.9300	C32—H32	0.9300
C13—C14	1.390 (4)	F3—P	1.570 (3)
C13—H13	0.9300	F6—P	1.566 (3)
C14—N2	1.377 (4)	N1—H1A	0.8600
N3—C20	1.317 (4)	N1—H1B	0.8600
N3—C16	1.331 (4)	N2—H2A	0.8600
C16—C17	1.373 (5)	N2—H2B	0.8600
C16—H16	0.9300	O1—H1	0.8200
C17—C18	1.381 (4)	O3—H3A	0.8200
C17—H17	0.9300	O1W—H1W	0.7910
C18—C19	1.378 (4)	O1W—H2W	0.8864
C18—C21	1.487 (4)		
			
N1—C1—C2	121.3 (3)	C19—C18—C21	121.5 (3)
N1—C1—C7	120.6 (4)	C17—C18—C21	122.6 (3)
C2—C1—C7	118.0 (3)	C18—C19—C20	120.2 (3)
C3—C2—C1	121.1 (3)	C18—C19—H19	119.9
C3—C2—H2	119.5	C20—C19—H19	119.9
C1—C2—H2	119.5	N3—C20—C19	123.7 (3)
C2—C3—C4	121.2 (3)	N3—C20—H20	118.1
C2—C3—H3	119.4	C19—C20—H20	118.2
C4—C3—H3	119.4	C26—C21—C22	115.6 (3)
C6—C4—C3	117.9 (3)	C26—C21—C18	121.4 (3)
C6—C4—C5	119.7 (3)	C22—C21—C18	123.0 (3)
C3—C4—C5	122.4 (3)	C23—C22—C21	120.5 (3)
O2—C5—O1	121.4 (3)	C23—C22—H22	119.7
O2—C5—C4	123.7 (3)	C21—C22—H22	119.7
O1—C5—C4	115.0 (3)	N4—C23—C22	122.8 (3)
C7—C6—C4	121.1 (3)	N4—C23—H23	118.6
C7—C6—H6	119.5	C22—C23—H23	118.6
C4—C6—H6	119.5	C25—N4—C23	117.1 (3)
C6—C7—C1	120.8 (3)	N4—C25—C26	123.6 (3)
C6—C7—H7	119.6	N4—C25—H25	118.2
C1—C7—H7	119.6	C26—C25—H25	118.2
C9—C8—C14	120.9 (3)	C21—C26—C25	120.4 (3)
C9—C8—H8	119.5	C21—C26—H26	119.8
C14—C8—H8	119.5	C25—C26—H26	119.8
C8—C9—C10	120.9 (3)	C32—N5—C28	119.5 (3)
C8—C9—H9	119.5	C32—N5—H27	120.3
C10—C9—H9	119.5	C28—N5—H27	120.3
C12—C10—C9	118.3 (3)	N5—C28—C29	121.8 (3)
C12—C10—C11	122.5 (3)	N5—C28—H28	119.1
C9—C10—C11	119.2 (3)	C29—C28—H28	119.1
O4—C11—O3	121.3 (3)	C30—C29—C28	120.5 (3)
O4—C11—C10	124.2 (3)	C30—C29—H29	119.8
O3—C11—C10	114.5 (3)	C28—C29—H29	119.8
C13—C12—C10	120.8 (3)	C29—C30—C31	115.9 (3)
C13—C12—H12	119.6	C29—C30—C30^i^	122.2 (3)
C10—C12—H12	119.6	C31—C30—C30^i^	121.8 (4)
C12—C13—C14	121.0 (3)	C32—C31—C30	120.7 (4)
C12—C13—H13	119.5	C32—C31—H31	119.6
C14—C13—H13	119.5	C30—C31—H31	119.6
N2—C14—C13	121.2 (3)	N5—C32—C31	121.6 (4)
N2—C14—C8	120.7 (3)	N5—C32—H32	119.2
C13—C14—C8	118.1 (3)	C31—C32—H32	119.2
C20—N3—C16	116.4 (3)	C1—N1—H1A	120.0
N3—C16—C17	123.5 (3)	C1—N1—H1B	120.0
N3—C16—H16	118.3	H1A—N1—H1B	120.0
C17—C16—H16	118.3	C14—N2—H2A	120.0
C16—C17—C18	120.3 (3)	C14—N2—H2B	120.0
C16—C17—H17	119.9	H2A—N2—H2B	120.0
C18—C17—H17	119.9	F6—P—F3	178.6 (2)
C19—C18—C17	115.9 (3)	H1W—O1W—H2W	115.1

Symmetry codes: (i) −*x*+2, −*y*, −*z*+1.

### Hydrogen-bond geometry (Å, °)

**Table:**

*D*—H···*A*	*D*—H	H···*A*	*D*···*A*	*D*—H···*A*
O1W—H2W···O4^ii^	0.89	1.87	2.751 (3)	169
O1W—H1W···O2^iii^	0.79	2.01	2.799 (3)	174
O3—H3A···N3^iv^	0.82	1.87	2.686 (3)	174
O1—H1···O1W^v^	0.82	1.80	2.617 (3)	173
N1—H1B···F3	0.86	2.57	3.324 (4)	147
N5—H27···N4	0.86	1.84	2.700 (4)	176

Symmetry codes: (ii) *x*−1, *y*, *z*; (iii) −*x*, −*y*+1, −*z*+1; (iv) *x*+1, *y*, *z*−1; (v) *x*, *y*, *z*+1.

## References

[bb1] Bruker (2004). *APEX2* and *SAINT* Bruker AXS Inc., Madison, Wisconsin, USA.

[bb3] Hu, D. H., Huang, W., Gou, S. H., Fang, J. L. & Fun, H. K. (2003). *Polyhedron*, **22**, 2661–2667.

[bb4] Sheldrick, G. M. (2008). *Acta Cryst.* A**64**, 112–122. 10.1107/S0108767307043930 18156677

[bb5] Yang, G. P., Wang, Z. Y. & Chen, J. T. (2004). *J. Mol. Struct.* **707**, 223–229.

